# Visual evoked potentials as a method for the prospective assessment of tacrolimus neurotoxicity in patients after kidney transplantation

**DOI:** 10.1007/s10633-022-09898-4

**Published:** 2022-10-26

**Authors:** Sebastian Sirek, Aureliusz Kolonko, Dorota Pojda-Wilczek

**Affiliations:** 1 Department of Ophthalmology, Faculty of Medical Sciences, Kornel Gibiński University Clinical Centre, Medical University of Silesia, Ceglana 35, 40-514 Katowice, Poland; 2 Kornel Gibiński University Clinical Centre, Katowice, Poland; 3 Department of Nephrology, Transplantation and Internal Medicine, Faculty of Medical Sciences, Medical University of Silesia, Katowice, Poland

**Keywords:** VEP, Calcineurin inhibitors, Kidney transplant, Neurotoxicity

## Abstract

**Introduction:**

Neurotoxicity, including optic nerve injury, is one of the most common adverse effects of tacrolimus, the principal calcineurin inhibitor used after kidney transplantation (KTx). The electrophysiologic measurements of both pattern visual evoked potentials (PVEP) and flash visual evoked potentials (FVEP) are valuable when drug-induced optic neuropathy is suspected.

**Objectives:**

To determine whether VEP measurement is a sensitive and repeatable method for monitoring tacrolimus neurotoxicity.

**Material and methods:**

This prospective study focused on 35 patients (20 M, 15F, 69 eyes, mean age 43 ± 11 years) who were at a median of 3.0 (IQR, 2.2–3.7) months after KTx at the time of the initial VEP evaluation and were treated with tacrolimus since KTx. The follow-up VEP examination was done after a median of 24 (22–27) months (both VEP measurements followed the ISCEV standards). The P100 wave latency and amplitude for the 1° and 15’ PVEP simulations, and the P2 wave latency and amplitude for the FVEP were analyzed.

**Results:**

For the 1° checks, the P100 wave latency and amplitude values were significantly worse in the follow-up examination compared to the early post-transplant time-point. Independent associations between FVEP parameters and the tacrolimus blood trough level were observed in the follow-up examination but not at the early post-transplant period. The P2 wave latency correlated with the tacrolimus trough level only in patients treated with the twice-daily, but not the once-daily, tacrolimus formulation. The brain derived neurotrophic factor (BDNF) level correlated with the P100 (15’) latency (*R* = 0.499; *p* = 0.005) and the P2 latency (*R* = 0.409; *p* = 0.025) only in patients treated with the once-daily, but not the twice-daily, tacrolimus formulation.

**Conclusion:**

The observations in this study may support the rationale for the use of VEP measurements as non-invasive monitoring of subclinical tacrolimus neurotoxicity.

## Introduction

Chronic kidney disease (CKD) is classified as a civilization-related disease and presents a challenge for healthcare worldwide. It is estimated that over 4 million adult Poles suffer from CKD [1]. Because the condition usually leads to irreversible end-stage renal disease (uremia) requiring permanent dialysis therapy, kidney transplantation is the optimal method of treatment [2]. Long-term function of the transplanted organ is warranted by the immunosuppressive treatment, most often based on a three-drug regimen, including calcineurin inhibitors (CNIs), an anti-metabolic drug and glucocorticosteroids. Since the 1980s, the indispensably used CNIs have been cyclosporine A and tacrolimus, which block the production of interleukins (IL), specifically IL-2, IL-3, IL-4, IL-5, and other cytokines, such as tumor necrosis factor *α* (TNF-*α*) and interferon gamma (IFN-*γ*) [3], i.e., compounds necessary to coordinate the functions of immune system cells. At present, tacrolimus is the main CNI agent used in kidney transplantation [4]. However, it also presents a significantly greater potential for neurotoxicity compared with cyclosporine A [5].

Due to the large inter-individual variability in its absorption and metabolism, tacrolimus is dosed individually based on the current concentration measured in the whole blood. However, except for the most frequently observed adverse effects, such as nephrotoxicity, diabetes mellitus, and infections, tacrolimus can also cause neurotoxicity [6, 7]. Among all immunosuppressive regimens, CNIs are particularly associated with drug-induced damage to the nervous system [7], because their lipophilic metabolites can penetrate the blood–brain barrier through damaged capillaries and accumulate in the brain tissue and cerebrospinal fluid [8, 9]. The mechanism of neurotoxicity is not fully understood and possibly involves oligodendrocytes, glia, and vascular endothelium injury [10]. The clinical spectrum of tacrolimus-induced neurologic complications is wide, including headache, tremor, paresthesia, and photophobia, as well as confusion, dysarthria, vision loss, and encephalopathy [6, 11, 12]. Importantly, the peripheral nerve dysfunction caused by tacrolimus may also occur with the blood trough levels within the therapeutic range [12]. The diagnosis of toxic optic neuropathy is usually based on a detailed medical history, laboratory tests, and a thorough eye examination. A complete ophthalmologic examination to assess suspected toxic optic neuropathy should include: visual acuity, color vision, and visual field tests; magnetic resonance imaging of the head and electrophysiologic tests. The latter are particularly useful in detecting subclinical toxicity.

## Aim

The purpose and main outcome of this study was the assessment of the quantitative changes in visual evoked potentials (VEP) during long-term neurotoxic tacrolimus-based therapy after kidney transplantation. Additionally, potential explanatory factors for the observed VEP changes were analyzed, including several laboratory parameters.

## Patients and methods

### Study group

Patient recruitment and laboratory measurements were carried out during a routine visit in the outpatient transplant clinic at the Department of Nephrology, Transplantation and Internal Medicine of the Medical University of Silesia in Katowice. Patients who had recently undergone transplantation and had stable renal excretory function qualified for the study, which was done in accordance with the Declaration of Helsinki. The Bioethics Committee of the Medical University of Silesia granted permission for the study protocol (KNW/0022/KB1/93/13).

The inclusion criteria were: age between 20 and 60 years, written informed consent, visual acuity ≥ 0.5 (decimal) on the Snellen chart, and treatment with one pharmacologic form of tacrolimus from the transplantation procedure to the time of the present study. The exclusion criteria included: absence or withdrawal of consent at any stage of the study, photosensitive epilepsy or other central nervous system disease (stroke condition, previous neurosurgical intervention, or major head injury), pre- or posttransplant diabetes, history of optic nerve (n. II) and/or retina diseases, cataract worsening visual acuity, nystagmus, photophobia or other unpleasant sensations caused by flashes of light, and treatment with other potentially neurotoxic drugs.

Both VEP examinations were done in 35 out of the 43 patients recruited into the study. Table 1 shows the clinical characteristics of study group during the baseline and follow-up examination. All study patients were treated with orally given tacrolimus during the whole period after kidney transplantation. At transplantation, all patients received the twice-daily tacrolimus formulation (Prograf). During the observation period of the study, 15 patients were converted from the twice-daily to the once-daily tacrolimus formulation (Advagraf) within a few months after the baseline examination. The main study outcomes were further analyzed separately for the different tacrolimus formulations.

**Table 1:** Clinical characteristics of study patients (n = 35) during baseline and follow-up study examinations

Parameter	First examination	Follow-up examination	*p*
Age [years]	40.9 (35.2–51.5)	42.9 (37.1–53.5)	
Sex [M/F]	20/15	−	−
Dialysis vintage [months]	24 (15–36)	−	−
BMI [kg/m^2^]	24.6 (21.0–27.9)	26.0 (23.0–29.5)	< 0.001
Time after transplantation [months]	3.0 (2.2–3.7)	27.8 (25.5–29.4)	
Serum creatinine [mg/dl]	1.4 (1.2–2.0)	1.3 (1.2–1.6)	< 0.01
eGFR [ml/min/1.73m^2^]	65.7 (47.2–77.5)	67.9 (58.2–85.9)	< 0.01
Tacrolimus daily dose [mg]	6.0 (4.5–8.0)	4.0 (3.0–4.5)	< 0.001
Tacrolimus dose per kg of body weight	0.09 (0.07–0.11)	0.05 (0.04–0.07)	< 0.001
Tacrolimus trough concentration [ng/ml]	10.3 (8.3–11.9)	6.3 (5.7–7.6)	< 0.001
Intraocular pressure [mmHg] for the right eye	16 (14–17) range 11–22	16 (15–18) range 10–24	0.06
Intraocular pressure [mmHg] for the left eye	16 (14–17) range 11–22	16 (15–18) range 13–20	0.43
Hs-CRP [mg/l]	2.2 (1.5–7.9)	2.7 (1.5–7.2)	0.52
IL-6 [pg/ml]	2.6 (1.7–3.7)	2.0 (1.3–2.7)	0.25
BDNF [pg/ml]	1320 (720–3740)	1720 (1080–4140)	0.28
ET-1 [pg/ml]	15.7 (12.0–29.0)	16.7 (10.7–21.2)	< 0.05
ADMA [ng/ml]	0.85 (0.62–1.40)	1.10 (0.90–1.40)	0.07

Data presented as median values with interquartile ranges. The Wilcoxon signed−rank test was applied. BMI, body mass index; VEP, visual−evoked potentials; eGFR, estimated glomerular filtration rate; hs−CRP, high sensitivity C−reactive protein; IL−6, interleukin−6; BDNF, brain−derived neurotrophic factor; ET−1, endothelin−1; ADMA, asymmetric dimethylarginine

The rest of the immunosuppressive regimen consisted of mycophenolate mofetil (orally, 500 mg BID) or sodium (orally, 360 mg BID) and steroids (prednisone, 7.5 mg during the baseline and 5 mg during the follow-up examination).

During the routine laboratory test, additional blood samples were withdrawn for the determination of several biochemical parameters and for the calculation of tacrolimus exposure by using the area under the curve (AUC). The AUC for tacrolimus was calculated with the formula:

AUC = 13.17 + (5.43 × “Tc0”) + (4.72 × “Tc3”).

where Tc0 is the tacrolimus blood level before the morning dose (trough level) and Tc3 is the tacrolimus blood level measured 3 h after the morning dose ingestion [13]. In the follow-up examination, only the tacrolimus trough level was measured.

Kidney graft function was measured by using an estimated glomerular filtration rate (eGFR) calculated according to the Modification of Diet in Renal Disease (MDRD) formula.

The historic control group, which consisted of patients who met the medical criteria of the present study but had no history of chronic kidney disease, was also included. In all control subjects, as the prospective study group, VEP measurements were done twice in the same laboratory. The control group included 15 subjects (27 eyes) with normal kidney excretory function, for whom the results of 2 consecutive VEP measurements were available. The median age was 52 (interquartile range, IQR, 46–66) years and the time between VEP examinations was 24 (19–31) months.

### Laboratory measurements

The laboratory measurements included the assessment of plasma concentrations of high-sensitivity C-reactive protein (hsCRP), IL-6, and brain derived neurotrophic factor (BDNF), as well as asymmetric dimethylarginine (ADMA) and endothelin-1 (ET-1). Table 2 provides detailed information on commercially available ELISA kits. All parameters were assessed twice, concomitantly with the baseline and follow-up VEP examinations.

**Table 2:** The characteristics of laboratory methods used in the present study

Measure	Method	LoQ	Intra-assay variation	Inter-assay variation
Hs-CRP	ELISA (Immundiagnostic AG, Bensheim, Germany)	0.09 mg/L	< 6%	< 11.6%
IL-6	ELISA (R&D Systems, Minneapolis, Minnesota)	0.7 pg/mL	< 4.3%	< 6.4%
BDNF	ELISA (R&D Systems, Minneapolis, Minnesota)	20 pg/mL	< 6.3%	< 11.4%
ADMA	ELISA (Immundiagnostik, AG, Bensheim, Germany)	0.16 μmol/L	< 7.6%	< 4%
ET-1	ELISA (USCN Life Sciences, Wuhan, China)	2.71 pg/mL	< 10%	< 12%

LoQ, limit of quantification; hs−CRP, high sensitivity C−reactive protein; IL−6, interleukin−6; BDNF, brain−derived neurotrophic factor; ADMA, asymmetric dimethylarginine; ET−1, endothelin−1

### VEPs and eye examinations

**Fig. 1:**
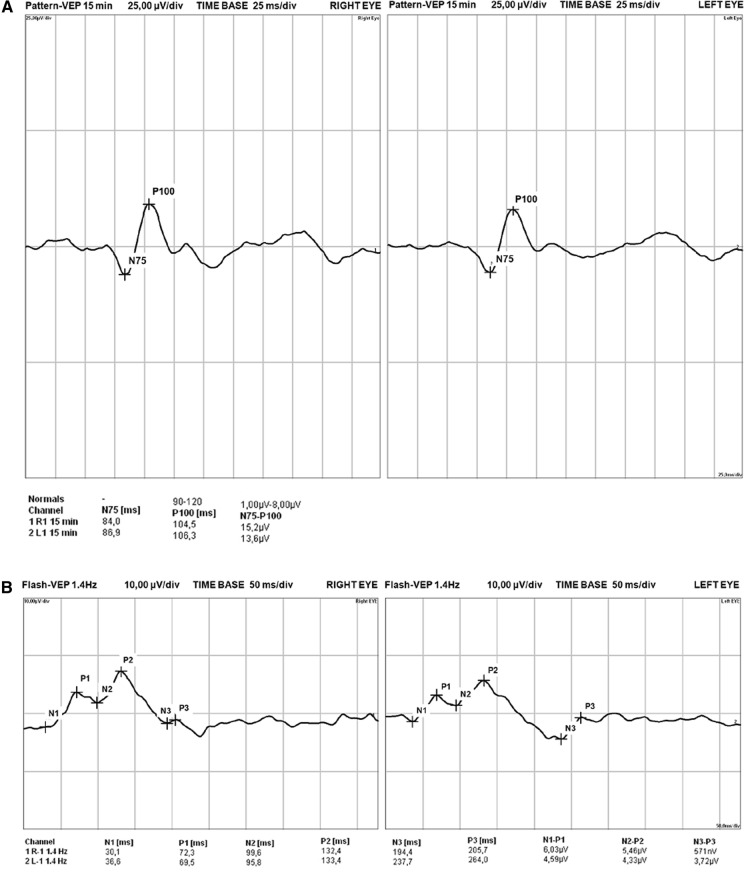
An exemplary trace of baseline pattern VEP (**a**) and flash VEP (**b**) measurements in kidney transplant recipient

General ophthalmologic examinations were done at the nearby Professor K. Gibiński University Clinical Centre of the Medical University of Silesia in Katowice. The best-corrected vision acuity was measured by using the Snellen chart. Patients with a defect above 3 dioptres were excluded. The first study examination (I) was carried out after the patient qualification, and the second examination (II) was done after a mean period of 25 (range, 21–28) months. Both examinations were carried out according to the International Society for Clinical Electrophysiology of Vision (ISCEV) standards, with minor variations in luminance and recording filters. Table 3 shows the detailed technical data on the VEP measurements; all study variables were measures twice. Visual evoked potentials (VEP) were tested by using the Reti-Port electrophysiologic device from Roland Consult (Germany), in accordance with the standards of the ISCEV [14]. The patient’s scalp was glued with gold cup electrodes by using abrasive paste (Nuprep Skin Prep Gel, Weaver and Company) and conductive paste (Ten20 Conductive Paste, Weaver and Company), according to the international system 10/20: the active electrode located at the Oz point (above the occipital area) in relation to the electrode at the Fz point (above the frontal area). The ground electrode was glued at point Cz (above the parietal area). A black-and-white checkerboard pattern with alternating phase change (pattern VEP; PVEP) was used to elicit a visual cortical response. Each eye of the patient was stimulated with a small (0.25 degree) and a large (1 degree) angular standard. For flash stimulation, (flash VEP; FVEP), standard flashes with a frequency of 1.4 Hz were used in the Ganzfeld stimulator. The amplitude and latency of the P100 PVEP wave (Fig. 1a) and P2 FVEP wave (Fig. 1b) were analysed. The amplitude was measured from the top of the negative peak preceding the positive (P) wave (N75-P100 and N2-P2), and the latency from the beginning of the stimulation to the occurrence of the highest value of the marked wave.

**Table 3:** The VEP measurement technical parameters

Parameter	Pattern VEP	Flash VEP
Stimulus mean luminance	80 cd/m^2^	3.0 cd/m^2^
Distance between eye and monitor	1 m	On the Ganzfeld
Visual angle	8.5 deg	−
Flash strength	−	3.0 cds/m^2^
Impedances	2−5 kOhm	2−5 kOhm
Signal amplification	50,000	50,000
Cut-off frequencies	Low Cut 1 Hz High Cut 50 Hz	Low Cut 0.5 Hz High Cut 50 Hz
Signals sampling	1,703 kHz	1,065 kHz
Recoding period and points	300 ms/512 points	480 ms/512 points
Amount of recording episodes	average 100 standard	average 100 standard

Each VEP test was preceded by checking of the visual acuity of the distance in the patient’s correction with glasses using Snellen charts and an intraocular pressure (IOP) test using a Goldmann applanation tonometer (CSO, Italy), and then finished with anterior and posterior examination of the eyeball in a slit lamp (Haag-Streit) by an indirect method using a Super Field lens (Volk, US) to monitor possible pathologic changes in the eye that may affect the result of the VEP test or the result of neurotoxicity (primarily changes in the optic disc, such as pallor and blurring or raising boundaries, etc.). Before the fundus examination, short-acting (4–6 h) mydriatics were given: parasympatholytic (1% Tropicamidum; Polfa) or sympathomimetic (10% Neosynephrin-POS; URSAPHARM) drops. If the patient did not consent to mydriasis, the fundus was checked through the narrow pupil. Refusal of consent for pupil dilation did not affect the patient's further participation in the study and did not change the patient’s treatment.

### Data analysis

The results of VEP measurements were scored according to the reference values (ISCEV) and expressed as percentages of the study subjects, data for the right and left eyes were combined and then individually analyzed. The primary outcomes of the present study were the relative changes in VEP parameters from the baseline values. Additionally, explanatory analyses were carried out on the variability of VEP measurements, including age, eGFR, tacrolimus level, and laboratory parameters.

### Statistical analysis

Data were collected on a Microsoft Excel 2007 spreadsheet and then analyzed by using the STATISTICA 13.3 software package for Windows (TIBCO Software Inc., Palo Alto, CA, USA) and MedCalc version 19.2.1 (MedCalc Software, Mariakerke, Belgium). The distribution normality was analyzed for all variables with the use of the Shapiro–Wilk test. All study data (except the mean visual acuity) were presented as median values with an interquartile range. Statistical calculations were done by using the Mann–Whitney U test and the Wilcoxon signed-rank test for variables with a non-normal distribution. The Spearman’s correlation coefficient R was used to assess the level of relationship between different variables. At the follow-up examination, further analyses were done separately for the two different tacrolimus formulations: twice-daily and once-daily tablets. Stepwise multiple regression analyses were performed for the P100 wave (1°) and (15^’^) latencies and the corresponding amplitudes as dependent variables, including age, eGFR, and log tacrolimus trough level as potential independent variables. Analogic regression analyses were done for the P2 wave latency and amplitude. The second model was used for the P100 (15’) latency and amplitude and P2 amplitude as dependent variables, including age, eGFR, log tacrolimus trough level, and log BDNF plasma level. The level of statistical significance in all analyses was set at p < 0.05.

## Results

### General ophthalmologic examination

The mean visual acuities on a decimal scale were 0.974 (95%CI, 0.952–0.996) and 0.920 (0.920–0.872) for the right and left eye, respectively, in the first examination, and 0.969 (0.947–0.991) and 0.912 (0.864–0.959) in the follow-up examination. No statistical differences were observed in the comparative test for visual acuity in both study examinations. In individual cases (5.8%), visual acuity decreased over a two-year observation period by a maximum of one line on the Snellen chart.

There were no statistically significant differences between the IOP measurements in the first and in the follow-up examinations (Table 1). In the assessment of the anterior eye segment structures, pathologic changes were observed in only one patient in the entire study cohort. During the follow-up examination, posterior subcapsular cataract was detected in both eyes, with long-term chronic kidney disease and pretransplant steroid therapy considered to be the most likely cause. The image of the eye fundus for the whole group did not differ from the norm. During the two-year follow-up, the patients did not complain of any eye symptoms.

### Tacrolimus dosing and levels

As expected, the tacrolimus daily dose and trough levels were significantly lower during the follow-up study examination than in the early post-transplant period (Table 1). Notably, there were no significant differences in tacrolimus dosing and trough levels between the patients treated with the two different tacrolimus formulations (data not shown).

### VEP measurements

At baseline, the median differences in PVEP P100 latencies between a given patient’s eyes were: 2.3 (IQR, 0.6–5.3) ms (1° checks), 3.0 (1.0–5.2) ms (15’ checks); for amplitudes, median interocular differences were 1.7 (1.0–2.6) μV (1°), and 1.8 (0.9–3.0) μV (15’), with maximum intrapatient differences of 15.3 and 17.6 ms, and of 9.8 and 10.7 μV, respectively. In FVEP, the median baseline differences were 5.2 (IQR, 1.9–7.6) ms and 1.8 (1.3–3.2) μV, respectively.

**Fig. 2:**
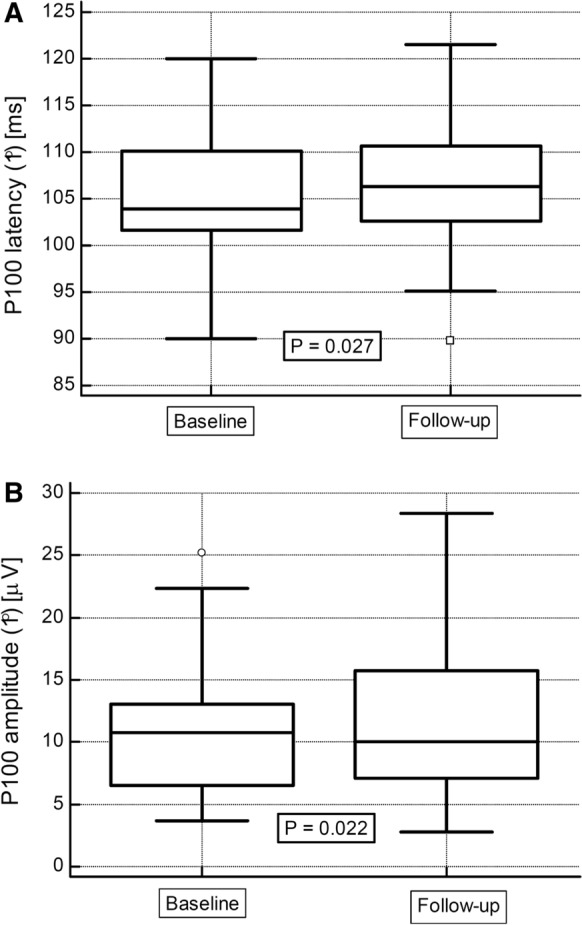
PVEP examinations in kidney recipients: median P100 latency (1°) (**a**) and median amplitude (1°) values (**b**) measured in the early post-transplant period (baseline) and after 24 months (follow-up). Wilcoxon signed-rank test. Median values with interquartile range (*box*) and non-outlier values (whiskers) are shown

In the PVEP examinations, in the study group, the follow-up latency (1°) was significantly longer than in the baseline post-transplant examination (Table 4, Fig. 2a). Additionally, a significant decrease of amplitude (1°) was observed during the same period (Fig. 2b). In contrast, there were no significant changes for the 15’ checks in P100 latency or amplitude values during the study period (Table 4).

**Table 4:** Pattern and flash VEP variables for baseline and follow-up examination in study group

	First examination	Follow-up examination	p
Pattern VEP examination – P100 parameters			
Latency (1°) [ms]	103.9 (101.6–110.0)	106.3 (102.7–110.4)	0.027
Latency (1°) > 116 ms [n (%)]	10 (14.5)	12 (17.4)	0.64
Latency (15’) [ms]	111.5 (108.6–118.6)	112.7 (107.4–118.0)	0.89
Latency (15’) > 116 ms [n (%)]	30 (43.5)	30 (43.5)	1.0
Amplitude (1°) [μV]	10.8 (6.6–13.0)	10.0 (7.1–15.7)	0.022
Amplitude (1°) < 10 μV [n (%)]	29 (42.0)	34 (49.3)	0.39
Amplitude (15’) [μV]	12.0 (7.8–16.7)	11.7 (8.7–16.2)	0.35
Amplitude (15’) < 10 μV [n (%)]	21 (30.4)	23 (33.3)	0.72
Flash VEP examination – P2 parameters			
Latency [ms]	109.0 (101.0–118.4)	109.9 (103.3–118.4)	0.78
Amplitude [μV]	8.8 (5.9–13.3)	7.5 (5.3–12.1)	0.25

Data presented as medians with interquartile range or frequencies. The Wilcoxon signed−rank test was applied. VEP, visual−evoked potentials

In the control group, no significant differences were observed between the baseline and follow-up PVEP measurements [median P100 latency (1°): 107.2 (IQR, 104.5–111.6) vs. 105.1 (102.2–107.4) ms; *p* = 0.16; median latency (15’): 109.8 (105.1–122.1) vs. 117.8 (112.7–123.0) ms; *p* = 0.40; median amplitude (15’): 8.1 (4.6–11.3) vs. 9.3 (5.3–17.2) μV; *p* = 0.20], except for amplitude (1°), for which the median value increased over time [7.5 (5.5–10.6) vs. 8.5 (5.9–9.4) μV; *p* < 0.05]. Thus, the latency (1°) and amplitude (1°) percentage changes in the control group were −1.9 and 13.5%, respectively. Similar longitudinal values of latency and amplitude were also found in FVEP examinations, both in the study and control groups. There were also no significant differences in the analyzed VEP parameters between the study and control groups, both at baseline and in the follow-up period.

At the first examination, P100 latencies were positively correlated between the check sizes (1° versus 15’) (*R* = 0.294; *p* = 0.015) but P100 was not correllated with P2 latency (*R* = 0.218; *p* = 0.072). P100 latency for the 1° checks was negatively correlated with (1°) and (15’) amplitudes (*R* = −0.487; *p* < 0.001 and *R* = −0.271; *p* = 0.025, respectively) and with P2 amplitude (*R* = −0.469; *p* < 0.001). There was a weak correlation between tacrolimus trough level and P2 wave latency (*R* = 0.241; *p* < 0.05). No other correlations were observed between either tacrolimus trough level or tacrolimus AUC and the other VEP parameters measured shortly after kidney transplantation. Notably, almost all VEP parameters (except P100 amplitude (15’) and P2 latency) showed a baseline association with recipient age, and all examined latencies, but not amplitudes, were much more pronounced in the follow-up examination (Table 5). However, in the stepwise multiple regression analysis, only eGFR (r_partial_ = 0.248; *p* < 0.05), but not age or log tacrolimus trough level, independently influenced P2 latency.

**Table 5:** The association between baseline and follow-up VEP parameters and recipient age

	First examination	Follow-up examination
Pattern VEP examination – P100 parameters		
Latency (1°) [ms]	*R* = 0.349; *p* = 0.003	*R* = 0.406; *p* < 0.001
Latency (15’) [ms]	*R* = 0.219; *p* = 0.07	*R* = 0.591; *p* < 0.001
Amplitude (1°) [μV]	*R* = −0.356; *p* = 0.003	*R* = −0.310; *p* < 0.01
Amplitude (15’) [μV]	*R* = −0.159; *p* = 0.19	*R* = −0.116; *p* = 0.34
Flash VEP examination – P2 parameters		
Latency [ms]	*R* = −0.025; *p* = 0.84	*R* = 0.220; *p* = 0.07
Amplitude [μV]	*R* = −0.427; *p* < 0.001	*R* = −0.370; *p* = 0.002

The Spearman rank test was applied

**Fig. 3:**
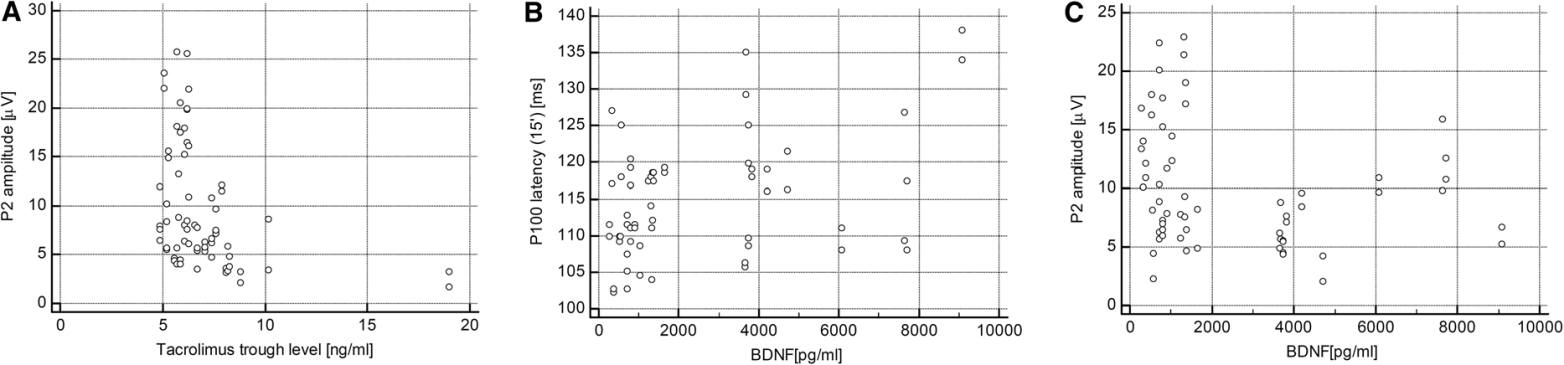
Association of P2 wave amplitude (**a**) with tacrolimus trough level in the follow-up examination, and associations of BDNF with P100 (15’) latency (**b**) and P2 wave amplitude (**c**) in baseline study examination. Calculated by using the Spearman correlation coefficient. For A: *R* = −0.450; *p* < 0.001, for B: *R* = 0.305; *p *= 0.017, for C: *R* = −0.304; *p* = 0.017

In the follow-up examination, there were only weak associations of P100 latencies between the check sizes (*R* = 0.276; *p* = 0.021) and between P100 for the 15' checks and P2 latency (*R* = 0.346; *p* < 0.01), whereas the correlations of (1°) amplitude with (15’) amplitude (*R* = 0.710); *p* < 0.001) and P2 wave amplitude (*R* = 0.512; *p* < 0.001) were much more pronounced. Notably, P2 wave amplitude, but not latency, correlated with tacrolimus trough level (*R* = −0.450; *p* < 0.001 and *R* = 0.183, *p* = 0.13, respectively) (Fig. 3a). Interestingly, when separate analyses were done for the two tacrolimus formulations, both the above associations were observed only in the twice-daily formulation subgroup (for latency *R* = 0.393; *p* = 0.013, and for amplitude *R* = −0.375; *p* = 0.019), whereas in the once-daily formulation subgroup, only amplitude (*R* = −0.480; *p* = 0.007), but not latency (*p* = 0.43), correlated with tacrolimus level. In the whole study group, the stepwise multiple regression analyses showed that P2 wave latency was independently influenced only by log tacrolimus trough level (*r*_partial_ = 0.472; *p* < 0.001), whereas P2 wave amplitude was affected by log tacrolimus trough level (*r*_partial_ = −0.420; *p* < 0.001) and age (r_partial_ = −0.404; *p* < 0.001).

In the analysing of inflammatory markers, IL-6 values significantly correlated only with P100 (15’) latency in the first (*R* = 0.300; *p* = 0.02), but not in the follow-up, examination.

The significant baseline association between BDNF plasma level and patient age (*R* = 0.457; *p* < 0.001) was not found in the follow-up examination (*p* = 0.98). Early after transplantation, there was a positive correlation of BDNF with P100 (15’) latency (*R* = 0.305; *p* = 0.017) (Fig. 3b) and a negative correlation with P2 wave amplitude (*R* = −0.304; *p* = 0.017) (Fig. 3c). In the whole study group, there was no significant association between BDNF and any VEP parameter in the follow-up measurement. However, there were significant positive correlations of BDNF with P100 (15’) latency (*R* = 0.499; *p* = 0.005) and P2 latency (*R* = 0.409; *p* = 0.025) only in patients treated with the once-daily, but not the twice-daily, tacrolimus formulation. Stepwise multiple regression analysis showed that log BDNF (*r*_partial_ =−0.383; *p* = 0.03) and eGFR (*r*_partial_ =−0.371; *p* = 0.004) were independently associated with P100 (15’) amplitude. Age and log tacrolimus trough level were not included in the final model.

In the follow-up examination, P100 (1°) latency and (1°) amplitude were associated with ET-1 levels (*R* = 0.299; *p* = 0.013 and *R* = −0.263; *p* = 0.03, respectively), but not with tacrolimus blood trough level.

In both examinations, there were no significant associations of VEP parameters with ADMA.

## Discussion

The present study described some observations that may confirm the utility of VEP measurements for non-invasive monitoring of tacrolimus neurotoxicity. First, both the P100 (1°) latency and (1°) amplitude values were significantly worse (later and smaller) in the follow-up examination compared with the early post-transplant time point. Second, independent associations between FVEP parameters and log tacrolimus blood trough level were observed in the follow-up examination but not in the early post-transplant period. Interestingly, P2 wave latency correlated with tacrolimus trough level only in patients treated throughout with the twice-daily formulation but not in those converted to the once-daily formulation.

VEP is a recorded occipital cortical evoked potential in response to visual stimulation [15]. PVEP abnormalities observed in uremic patients include delayed peak latency and reduced amplitude of P100 component [16, 17], which decreased after the hemodialysis session [18]. In the 1970s and 1980s [16, 19] but not in the modern era [20], normalization of both P100 latency and amplitude was reported after successful kidney transplantation, when uremia-induced metabolic disturbances subside. The lack of complete normalization of VEP recordings, also observed in the present study, may be attributed to the post-transplant optic nerve dysfunction caused by CNI neurotoxicity, which is further supported by literature reports of tacrolimus-associated neurotoxicity in liver transplant recipients without uremia [5, 21]. Besides, a higher prevalence of clinical neuropathy and nerve excitability parameters was found in kidney transplant recipients receiving CNI-based immunosuppression compared with a CNI-free regimen [15]. In this regard, a case of tacrolimus-associated brachial neuritis recovered only after tacrolimus cessation and replacement with everolimus [22]. It is worth noting that in a majority of uremic subjects, those abnormalities were detected without any pathologies in neurologic examination [23]. Similarly, reversible VEP changes secondary to early ethambutol toxicity were observed in patients with normal results of neuro-ophthalmologic examination [24, 25]. All the above mentioned evidence support the hypothesis that abnormal values of VEP parameters reflect the tacrolimus-induced subclinical changes in the bioelectrical function of the optic nerve.

If this is the case, a greater degree of VEP abnormalities should be observed in patients who have been on tacrolimus-based immunosuppressive therapy for a longer period. In the study group, both the P100 latency and amplitude stimulated with a large angular (1°) pattern were significantly worse in the follow-up examination, whereas both the (15’) latency and amplitude did not change over the period of observation. Because the (1°) VEP measures are particularly linked with the detection of subclinical optic neuropathy [26], the toxic effect of tacrolimus may seem to worsen with a longer therapy. Notably, such a time-dependent worsening of PVEP parameters was not observed in the control group without tacrolimus therapy. In this regard, an independent positive association of P2 wave latency and a negative association of P2 wave amplitude with log tacrolimus blood trough level were found in the follow-up examination, which was not observed in the early post-transplant period. Interestingly, increasing values of the correlation coefficients between increasing patient age and almost all analyzed VEP parameters were observed; however, age did not have an independent influence on the main study outcomes. These results may suggest that abnormal VEP values correlate instead with long-term chronic tacrolimus toxicity, which is also partly responsible for irreversible chronic kidney graft injury (i.e., tubular atrophy/interstitial fibrosis), the main cause of late graft function deterioration and loss.

Interestingly, there were significant differences in the latter associations depending on the pharmacologic formulation of tacrolimus, with the P2 latency positively correlated with tacrolimus level only in the twice-daily subgroup. In our earlier analysis of stable kidney transplant patients, both the type of tacrolimus formulation and drug exposure influenced VEP parameters [27]. Thus, both our previous and present observations may suggest a lower neurotoxic potential of the once-daily tacrolimus formulation. In fact, this formulation was developed to increase drug adherence, although lower toxicity was also anticipated due to its more flattened blood level slope with a significantly lower maximum concentration. In healthy volunteers, improvement of renal hemodynamics and function was observed with the once-daily extended release formulation [28]. Importantly, lower neurotoxicity assessed by the degree of tremor was recently reported with the use of another slow-release tacrolimus formulation [29].

To get some insight into the pathophysiologic mechanism of tacrolimus neurotoxicity, the plasma levels of BDNF were assessed at both VEP examinations. BDNF, a critical protein for the maintenance of neuronal functions, has been postulated to control neural development, modulate synaptogenesis, and have some neuroprotective effects [30]. Experimental studies have shown that tacrolimus stimulates the expression and release of BDNF in cultures of cortical astrocytes exposed to simulated ischemic conditions; however, the extent to which these effects contribute to neuroprotection in vivo remains unknown [31]. Notably, there was a significant decrease of BDNF expression in the hippocampus in control and diabetic rats receiving daily tacrolimus (1.5 mg/kg) injections for 6 weeks [32], with the most pronounced effect in diabetic rats. In the present study, BDNF level in the early post-transplant period was strongly associated with age and worse values of both VEP P100 component (15’) latency and P2 wave amplitude. Interestingly, the association of BDNF level with (15’) and P2 latencies in the follow-up observation was observed only in patients treated with the once-daily tacrolimus formulation. Concomitantly, the evident influence of tacrolimus exposure on several VEP parameters and BDNF level was noted. Based on these results, it cannot be dismissed that tacrolimus neurotoxicity in vivo may be mediated with BDNF.

The main limitation of this study is the low number of participants, mainly due to the strict exclusion criteria, which include any form of both pre- and post-transplant diabetes and previous neurologic abnormalities. It is worth noting that in the main cohort, except for approximately 20% of kidney recipients with preexisting diabetes transplanted during the study recruitment, another 20% was excluded due to the ongoing glucose metabolism disturbances; some other patients were excluded due to unfavorable data in their anamnesis. Additionally, because an age-related P100 latency increase was noted in patients older than 60 years [33], another 18% of potential study candidates were excluded. Several patients clearly satisfied more than one exclusion criterion. Moreover, the need for additional blood withdrawal and the inconvenience caused by the fact that VEP measurements had to be done in a distant medical ophthalmology center further limited the number of participants. Nevertheless, this is the first clinical study that aimed to evaluate the usefulness of VEP measurements in the monitoring of potential tacrolimus neurotoxicity. Another limitation is the lack of other vision function or morphology tests (i.e., Humphrey visual field, optical coherence tomography, or multifocal electroretinography) at the time of VEP examination, which could help to confirm the findings; this was due to time and organization constraints.

It is worth emphasizing that regular monitoring and detection of subclinical tacrolimus neurotoxicity by using VEP may be beneficial not only in the ophthalmologic setting, in which it offers the advantage of very sensitive recognition of neurotoxicity before visual alterations are detected by ophthalmologic exam. In future clinical practice, abnormal VEP results should motivate the modification of maintenance immunosuppressive therapy toward the less neuro- and nephrotoxic tacrolimus formulations or other immunosuppressants without such an undesirable potential. Such a maneuver, resulting in the minimization of long-term tacrolimus nephrotoxicity, could improve kidney graft survival in this specific subgroup of kidney transplant recipients.

## Conclusion

The present study showed that both P100 latency and amplitude values for 1° checkerboard reversal were significantly delayed and diminished, respectively, after 2 years of tacrolimus treatment compared with the early post-transplant period. During the third post-transplant year, both P2 wave latency and amplitude values were negatively associated with tacrolimus blood trough level. Moreover, P2 latency correlated with tacrolimus trough level only in patients treated with the twice-daily, but not the once-daily tacrolimus formulation. These observations may support the rationale for the use of VEP measurements as a valuable tool for non-invasive monitoring of suspected subclinical tacrolimus neurotoxicity. In this regard, abnormal VEP values provide strong support for immunosuppressive regimen modification, such as a decrease in the tacrolimus dose or conversion to the alternative, non-neurotoxic mTOR inhibitor therapy.

## Data Availability

The data that support the findings of this study are available from the corresponding author upon reasonable request.
